# Optimal growth conditions to enhance *Chlorella vulgaris* biomass production in indoor phyto tank and quality assessment of feed and culture stock

**DOI:** 10.1016/j.heliyon.2024.e31900

**Published:** 2024-05-24

**Authors:** Turfatul Jannat Jui, Anika Tasnim, S.M. Rashadul Islam, Omar Hamza Bin Manjur, Md. Saddam Hossain, Nishat Tasnim, Debabrata Karmakar, Md. Rakibul Hasan, Md. Rezaul Karim

**Affiliations:** Institute of Technology Transfer and Innovation, Bangladesh Council of Scientific and Industrial Research, Dhaka, 1205, Bangladesh

**Keywords:** light:dark photoperiod, Algal biomass, Microalgae, Feed source, *C. vulgaris*

## Abstract

Commercial microalgae cultivation is a dynamic field with ongoing efforts to improve efficiency, reduce costs, and explore new applications. We conducted a study to examine how different light exposure periods affect *Chlorella vulgaris's* growth. We employed a Phyto tank batch system of approximately 3.5 L with LED light control, controlled airflow, and sterilized bags, maintained at 22.0 ± 2.0 °C indoors. Various methods, including spectrophotometry, and cell counter were employed to monitor *Chlorella vulgaris* growth under different light exposure cycles. Additionally, quality analysis as feed source was employed by proximate, amino acid, beta-glucan, and microbial content analysis. The results revealed significant variations in *C. vulgaris* biomass production based on light exposure duration. Notably, the 16:8-h light-dark photoperiod exhibited the highest biomass concentration, reaching 6.48 × 10^7^ ± 0.50 cells/mL with an optical density (OD) of 1.165 absorbance at 682 nm. The 12:12-h light-dark photoperiod produced the second-highest biomass concentration, with 2.305 × 10^6^ ± 0.60 cells/mL at an OD of 0.489. Proximate analysis of dry algae powder revealed low lipid content (0.48 %), high protein content (37.61 %), variable ash concentration (average 10.75 %), and a significant carbohydrate fraction (51.16 %) during extended daylight and shorter dark periods. Amino acid analysis identified nine essential amino acids, with glutamic acid being the most abundant (17.7 %) and methionine the least (0.4 %). Furthermore, quality analysis and microbiological assays demonstrated that the *C. vulgaris* biomass is well-suited for fish and livestock use as a feed source and possibility as human nutraceuticals. These findings can be considered more environmentally friendly and ethically sound due to the absence of genetic modification.

## Highlights

• The 16:8-h light-dark photoperiod can increase biomass production.
• Nutritionally enriched microalgae powder makes it a valuable resource for the aquaculture industry.
• Essential amino acid profiling reveals it as a high-quality protein source.

## Graphical abstract

**Figure:**
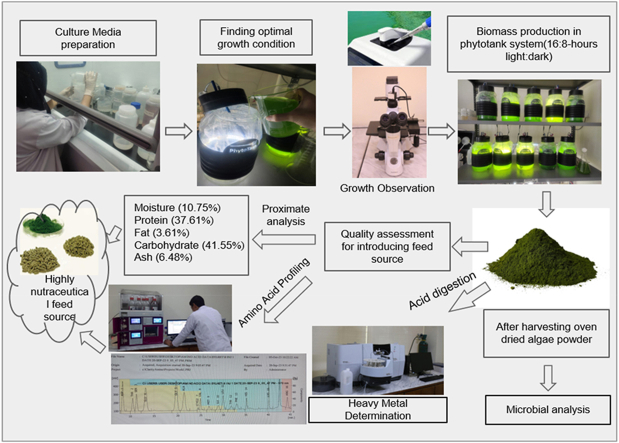

## 1 Introduction

*Chlorella vulgaris*, a green unicellular microalgae has drawn a lot of interest for its versatile uses including wastewater treatment, aquaculture feed, and biomass as a source of crucial chemical compounds [1]. Approximately 30 % of microalgal cultivation is allocated for animal feed applications, driven by the growing consumer preference for food products containing natural components rather than artificial additives [2]. This heightened demand has spurred substantial scientific investigation aimed at identifying natural components capable of enhancing the nutritional quality of animal-derived food items [3]. This species also provides essential energy and natural vitamins for the growth and improvement of larvae and juvenile aquatic invertebrates [4,5]. Its primary components consist of a distinctive and varied combination of essential macronutrients and micronutrients, encompassing proteins, omega-3 polyunsaturated fats, polysaccharides, vitamins, and minerals, contributing to overall health [5].

Additionally, research has shown that the consumption of supplements containing *C. vulgaris* can lead to a decrease in high levels of lipids and blood sugar, while also providing protection against oxidative stress, cancer, and chronic obstructive pulmonary disease [6]. It's treated as a high-quality protein source that may be readily absorbed by animals, with a high protein concentration of up to 60 % [7]. Microalgae, in general, have a high chlorophyll content and a fibrous outer cell, which helps to boost the amount of good bacteria in the digestive tract [5]. In addition to its widely recognized benefits as a food and nutrient source, chlorella has garnered significant attention in current research. Its rich nutritional profile positions it as a promising candidate for addressing various dietary and health needs. As researchers delve deeper into the mechanisms underlying chlorella's health-promoting properties, the stage is set for unlocking its full potential in nutrition and wellness [7,8].

Bulk microalgal production has gained considerable attention in various settings, as highlighted in studies by Brennan et al. (2010), Chisti (2008), and Sheridan (2009) [[8], [9], [10]]. However, it is important to note that the composition of *C. vulgaris*, including its color, unsaturated fatty acid, carbohydrate, and protein content, can vary with changes in light intensity, as discussed by Maltsev et al. (2021) [7]. Numerous research efforts have been dedicated to exploring the relationship between light intensity and environmental factors to support the widespread cultivation of microalgae. Factors like incubation temperature, nutrient levels, growth medium salinity, and light not only impact photosynthesis and cell biomass productivity but also influence cellular metabolism and composition [11,12]. While the first three factors can be reasonably optimized through human intervention, achieving ideal lighting conditions for mass microalgae cultivation remains a more challenging task.Light plays a crucial role as the primary energy source for microalgal photosynthesis. Firstly, there are light reactions, converting light energy into chemical energy. Secondly, dark reactions produce organic compounds like carbohydrates and lipids [13]. Excessive light can pose a threat to the functioning of photosystem II processes as it can limit the flow of electrons. This limitation in electron flow not only impedes the process of photosynthesis but also leads to a condition known as photo-inhibition, which can be quite stressful for organisms [14].

Indoor Phyto Tank systems present a promising avenue for cultivating *Chlorella vulgaris* on a large scale with precise control over growth parameters, such as light intensity, temperature, nutrient availability, and carbon dioxide concentration [5]. Optimizing these conditions in Phyto Tanks has the potential to enhance biomass production efficiency and maximize the accumulation of valuable bioactive compounds within the microalgae. In the photo-bioreactor, incident light passes through the microalgal growth surface and is absorbed along the route, resulting in a light gradient. It also transfers the algal cells between the saturating light zone of the photo-bioreactor and the dark interior of the culture. As a result, the algal cells go through predictable cycles of light and shade [15]. Numerous investigations have been conducted to comprehend how variations in light/dark cycles affect microalgal response [12]. The findings indicated that intermittent light, or brief light flashes separated by dark intervals, may allow for more efficient utilization of available light.

Recent research has focused on enhancing phytoplankton cultivation techniques to boost biomass yields and improve the nutritional profile of these microorganisms. Light intensity and duration play critical roles in phytoplankton growth, emphasizing the importance of optimal lighting conditions in cultivation processes [5,12]. While progress has been made, current biomass generation remains insufficient to satisfy market demand. *C. vulgaris* exhibits a variety of beneficial uses as feedstock to enhance microalgae biomass production and address challenges associated with harvesting [1]. Its efficient utilization of photosynthesis makes it a suitable candidate for addressing food security concerns, especially in early-stage aquaculture.

The study's main objective was to determine out how *C. vulgaris* responds to changes in photoperiod lengths and light intensity in a low-cost model photo-bioreactor simultaneously. Furthermore, the research aimed to assess the nutrient levels, amino acid composition, and microbial content within the produced dry algae powder.

## 2 Materials and methods

### 2.1 Biomass production of *Chlorella vulgaris*

#### 2.1.1 Sample collection and media preparation

Pure isolate *Chlorella* sp. seeds were obtained from the University of Texas at Austin's Culture Collection of Algae. Three replicas of *C. vulgaris* (UTEX 2714) were used in this experiment, and they were cultivated in a 500 ml conical flask with 150 ml of bold basal media according to Stein and J.R., (1973) [16].

To set up the phyto-tank, we use a see-through tank, good lighting, and a way to move the water around. The volume of phyto-tank is 3.5 L, and the integrated LED light provides uniform lighting for maximal growth and obviates the need for a space-consuming bank of fluorescent lights. A warm-white fluorescent lamp delivering 3000 Lux of illumination at varying light-dark cycle periods was used to incubate three replicates of the microalgae at 22**°**C ± 2**°**C [17,18]. The cultures tank was placed in disposable culture bags, and the Phyto-tanks durable rubber cap had bulkheads for disposable aeration and vent tubes added autoclave water to the halfway point, added 250 ml of starter culture with media, installed lid and tightened clamp, then supplied air hose with inline filter, then plug power supply. Microalgae harvest within 7–10 days and dispose of the plastic bag liner, vent, and aeration tubes.

#### 2.1.2 Mass culture in a phyto tank system

Five milliliters of pure microalgae from a stock culture were mixed with 15 mL of Bold Basal culture medium in a 50-mL conical flask. This mixture was then cultivated continuously. An air filter was placed at the top of a glass pipette, which was sealed with non-absorbent cotton wool to serve as an aerating tube. When the microalgae reached the stationary growth phase, 50 mL of the culture were transferred into 200 mL of culture medium in a 500-mL conical flask. This process was repeated to scale up the culture.

In a phytotank system, 300 mL of microalgae culture were mixed with 2000 mL of culture medium. The phytotank had 12 culture reactors, each with a 6-inch diameter and a working volume of 3.5 L. It featured an integrated LED light and was equipped with a silent air pump that circulated 100 L of air per hour.

An aeration hose was attached to the tank cap and left running for 24 h. An LED light was affixed to the tank body to provide continuous illumination during cultivation. After five days of inoculation, the desired amount of algae began to appear. The temperature fluctuated between 22 °C and 24 °C throughout the growth period.

#### 2.1.3 Determination of biomass concentration

Algal concentration was determined using an automated fluorescence cell counter and light microscope [19].To prepare the sample, the phyto tank required vigorous shaking for at least 30 s, and 1 mL of algae was extracted using a transfer pipette. A 10-μL algal sample, with the number of cells determined by compound microscope counting, was placed in slot A. The mean density and fluorescence dye concentration were measured with automated cell counters, distinguishing between live and dead cells and providing data on cell diameter, viability, and numbers. Cell density was calculated as Average cell * dilution factor * 10^4^. Uv vis spectrophotometer scan of the culture sample from 600 to 800 nm identified the peak absorbance at 682 nm, the most sensitive wavelength for *C. vulgaris* concentration measurement. This wavelength was subsequently used for all assessments of growth phase. Cell concentration was measured by an inverted biological microscope (Raxvision Y100, Malaysia), and the suspension's optical density was assessed using UV–vis-Spectrophotometer (Hach, DR-6000, India). Chlorella cell counting was conducted using the CellDrop™ Automated Cell Counters (deNovix Inc., USA).

Algal biomass was concentrated as steps of 5-min, 5000-rpm centrifugation on a manual centrifuge (Centrifuge 5804 R, Eppendorf, Germany). Samples were washed twice with deionized water and dried at 50°C–60 °C with 40 % humidity for 4 h.

This method usually entails spraying a binder liquid over fluidized particles, which, while wet, will bind the particles together through liquid bridges [20]. It might also require drying the mixture in a spray granulator. Depending on the type of binder used, the liquid bridge will either solidify with cooling or dry out with heating to produce a solid bridge.

### 2.2 Quality analysis

#### 2.2.1 Moisture content determination

The moisture content of cultivated algae samples was assessed using an infrared moisture analyzer (model MOC63u, Shimadzu, Japan).Three separate samples, each weighing 1000 g, were placed onto an aluminum plate and subjected to drying at 105 °C until a consistent weight was reached. The moisture content was determined by calculating the proportion of mass lost during this process.

#### 2.2.2 Protein determination

The Dumas combustion method was utilized to determine the concentration of nitrogen using a nitrogen analyzer (NDA 702, VELP, Italy) [21]. Samples are prior to oxidative digestion at high temperatures with a controlled oxygen supply. After undergoing reduction and carrier gas purification processes, the remaining nitrogen content was detected using a thermal conductivity detector. The electrical measurement signal generated by the N2 volume was used to measure and calculate the nitrogen content of the burned samples, based on a pre-prepared calibration curve.

#### 2.2.3 Lipid determination

The American Oil Chemists' Society (Revised 2017) approved technique for extracting fat, AOCS Ba 3–38, was used. The samples were extracted using the Fat Extractor (E−500 ECE, Buchi, Germany) as described by Twisselmann (1923) [22]. At first, each beaker was dried for 30 min at 102 °C. After that, it was weighed after cooling at room temperature in a desiccator for at least an hour.

#### 2.2.4 Ash determination

The ash content was determined by comparing the initial and final weights of the samples after they were subjected to a 24-h, 500 °C heating process. To calculate the total carbohydrate content, the following formula was used:

Total carbohydrates (% of the fresh weight) = 100 - moisture (% of the fresh weight) - protein content (% of the fresh weight) - crude fat (% of the fresh weight) - ash (% of the fresh weight) [23].

### 2.3 Amino acid analysis

Amino Acid Analyzer (S 433-D, Sykam AAA, Germany) was used to conduct this amino acid assay. The chromatographic technique was employed for this experiment. 250 mg of the sample was dissolved in 500 ml of hydrolysis solution, which was made up of 300 ml of 37 % HCL, 200 ml of DI water, and 0.5 g of phenol. The samples were then maintained at 120 °C for 24 h. The samples were filtered using Whatman no. 01 Filter Paper after incubation. The samples have to be prepared with buffer pH-adjustable 1 M & 7.5 M NaOH solutions. The sample's pH ranged from 2.9 to 3.1. Following pH adjustment, the sample was diluted to 250 mL, and 100 mL was then extracted from this stock, which was then further filtered using a 0.45-M syringe filter. The samples were then stored in the autosampler. The procedures outlined by Zhou et al., 2022 are used to complete the subsequent phases [24]. The samples are divided into the columns based on their charges and retention times. The quantity of protein in the samples was estimated after gathering all the data and running one standard solution in comparison to this standard curve.

### 2.4 β-glucan content determination

The dried algae powder was analyzed for its β-glucan content using the Megazyme assay kit designed for mixed linkage β-glucan analysis. This analysis involved the hydrolysis of the powder with lichenase (at a concentration of 1000 U/ml) and b-glucosidase (at a concentration of 40 U/ml). The resulting β-glucan concentration is expressed as a percentage of the total sample weight.

### 2.5 Heavy metal analysis

From the algae sample 0.5 g dry powder was taken in a 100 ml beaker and placed on the hotplate. Thereafter, 20 ml of concentrated HNO_3_ and 10 ml concentrated HClO_4_ acid was added and heated at 200**°** C (at nearly dry). Followed samples were allowed to cool at room temperature, and made the final volume with DI (Deionized water) in a 50 ml volumetric flask. Further dilutions were made as per required. Finally, the samples were examined with AAS (Atomic Absorption Spectrophotometer) (AA-7000, SHIMADZU, Japan) for metal estimation followed by respective Equipment Standard Operating Procedure [25].

### 2.6 Microbiological analysis

Plate Count Agar (PCA) (Hi media, India) and physiological saline solution (0.85 % NaCl) were used to perform aerobic plate count according to the methods given in Cowan and Steel's manual [26]. All tests had been conducted through a spread plating method after serial dilution (10 ^1^ -10 ^6^).

### 2.7 Statistical analysis

All the data were reported as mean ± SD using R Studio version 4.2.2 for final analysis. The mean values were calculated based on the data taken from at least three independent experiments using freshly prepared reagents.

## 3 Results

### 3.1 Biomass production of *C. vulgaris*

Green microalgae *C. vulgaris* were selected as viable potential strains to boost the biomass output in indoor phyto tank systems with increased artificial light. To establish the appropriate cell concentration based on absorbance maximum wavelength, illumination parameters, growth rate, and biomass production were evaluated at different photoperiods of cycles. The cell density of microalgae was measured using a Cell Counter under various photoperiod conditions.

**Fig. 1:**
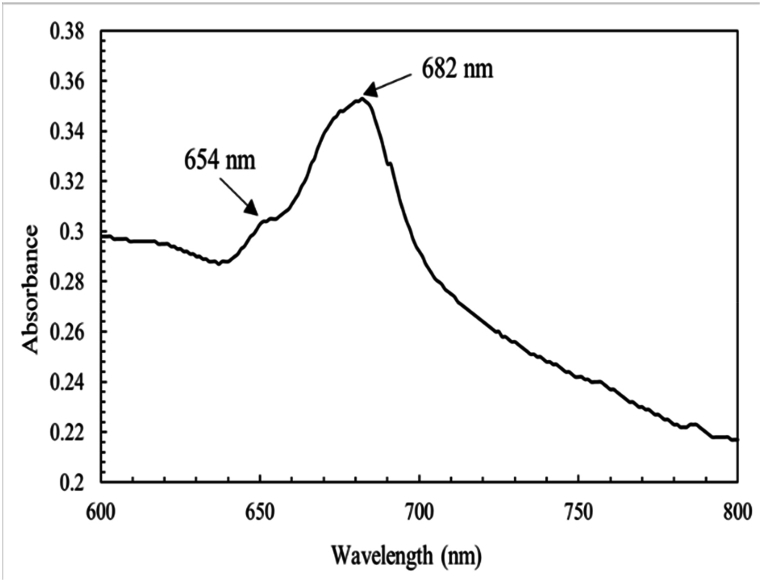
Light absorbance pattern for a solution with Chlorella vulgaris screened between 600 and 800 nm.

**Fig. 2:**
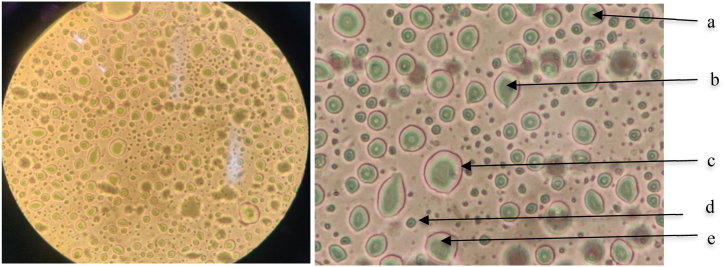
Microscopic observation of *Chlorella vulgaris* (40x magnification).

The previous experiment revealed that the ideal pH range for *C. vulgaris* growth was between 7.0 and 7.5 under optimal light intensity [27]. When the pH was maintained at 9 and 10, the pH of the culture suspension gradually decreased over several days [28]. A culture sample from 600 to 800 nm identified the peak absorbance at 682 nm, the most sensitive wavelength for *Chlorella vulgaris* measurement (Fig. 1). This wavelength was subsequently used for all assessments. Different absorbance responses were observed in these photoperiods when Chlorella species were grown in the Bold basal medium. To determine the best density, growth rate, and biomass output were assessed under three different light cycle: dark cycle photoperiods (Fig. 2).

**Fig. 3:**
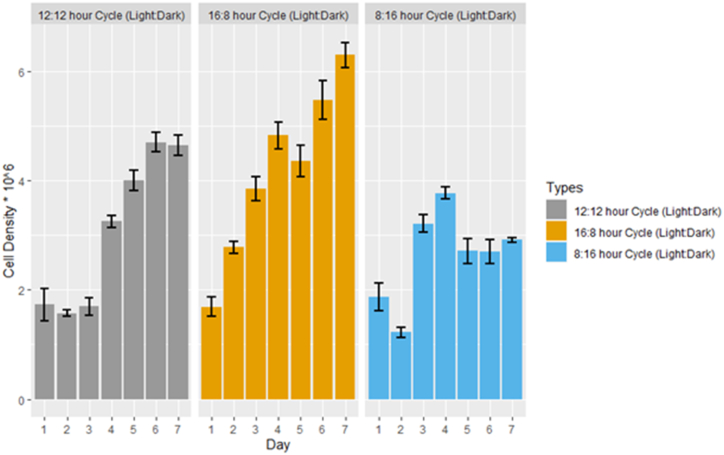
Cell density of *C. vulgaris* at three distinct photoperiods is depicted. The Y-axis indicates the cell count (Cell density*106), while the X-axis represents the number of days for each photoperiod. The mean cell density is greatest during the 16:8 h (light: dark) cycle and least during the 8:16 h (light: dark) cycle. The 16:8 cycle, denoted by the golden shade, exhibits a progressive daily increase in cell density that continues to peak on the seventh day of the existing cohort. The error bar in black indicates ± standard deviation (SD).

**Fig. 4:**
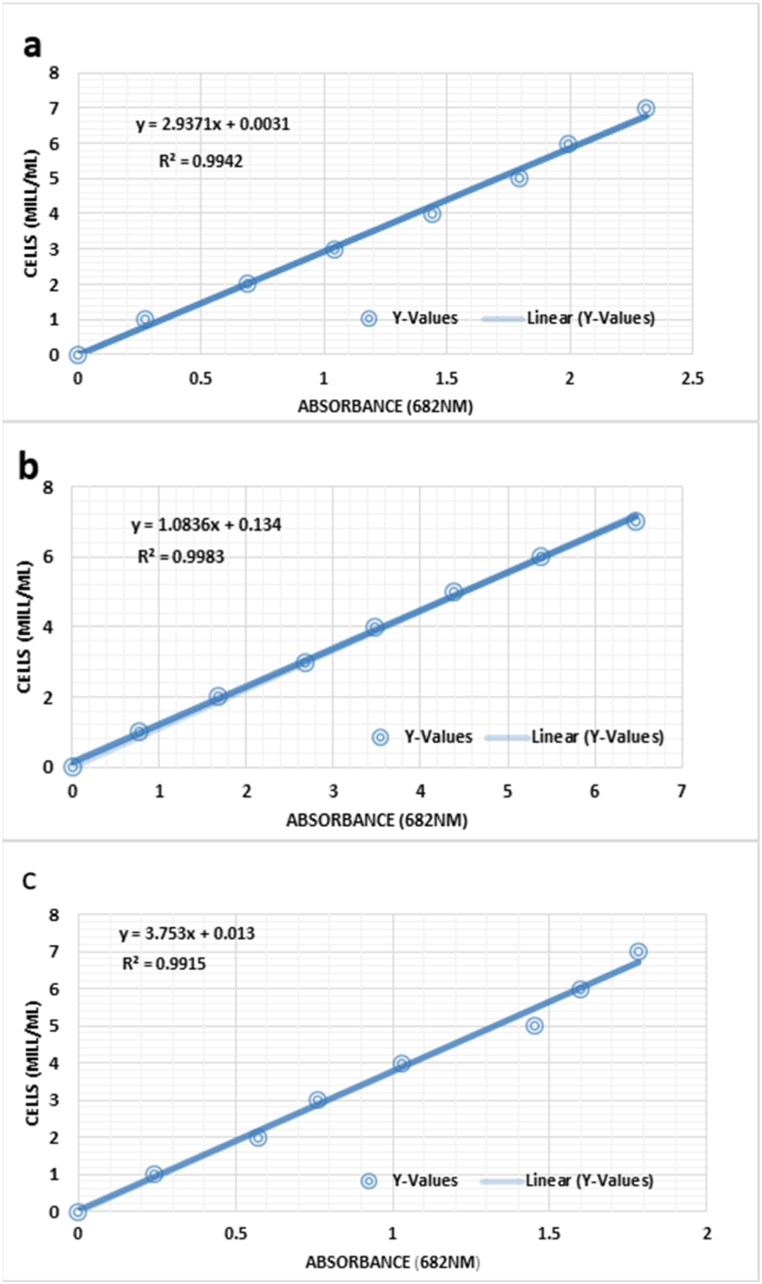
Relationship between absorbance (682 nm) and cell density for *C. vulgaris* at (a) 12:12 h, (b) 16:8 h, and (c) 8:16 h (light:dark cycle) Photoperiods.

The results in Fig. 3 clearly demonstrate that for *C. vulgaris*, a longer growth period with a 16:8-h light: dark cycle in the phyto-tanks culture technique leads to the highest cell density. Fig. 4a - c illustrates the relationship between absorbance and cell density for *C. vulgaris* under different photoperiod cycles. The line in the graph represents the absorbance equation for the microalgae growth curve: y = mx + c (R_2_ = 0.999), where y represents cell density (in millions) and x represents absorbance at 682 nm, m denotes the slope of the curve and c denotes the intercept of the y-axis.

**Fig. 5:**
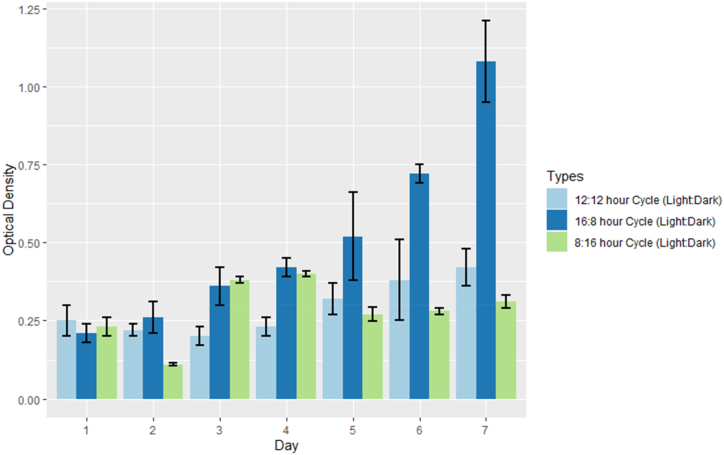
The biomass of *C. vulgaris* underwent three distinct photoperiods, as depicted. In the last three days of the 16:8 h Cycle (light:dark), the optical density has increased exponentially at 682 nm. The error bar in black indicates ± standard deviation (SD).

*C. vulgaris* exhibited the highest cell density of 6.4833 x 10^6^ cells/ml with an optical density (OD) of 1.165 absorbance at 682 nm in a 7-day culture period when exposed to a 16:8-h light:dark cycle. The 12:12-h light:dark photoperiod produced the second-highest biomass concentration, with 2.305 × 10^6^± 0.60 cells/mL at an OD of 0.489. The 8:16-h light:dark photoperiods resulted in a biomass concentration of 1.783 × 10^6^± 0.50 cells/mL at an OD of 0.312. after 7 days of cultivation (Fig. 5).

### 3.2 Proximate analysis

Table 1 presents the composition of total lipids, proteins, ash, and carbohydrates in *Chlorella vulgaris* dry powder. Only a minimal amount, specifically 0.48 %, of total lipids was found, which is relatively low compared to other algae species like Dunaliella and Scenedesmus species. The protein content in the investigated algae species was notably high, accounting for approximately 37.61 %, surpassing levels observed in other algae species. The ash concentration in these algae species exhibited significant variation, averaging at 10.75 %. After subtracting, the remaining composition was 51.16 %, presented as the total carbohydrate content.

**Table 1:** Proximate composition of dried *C. vulgaris*.

Sample Name	Moisture %	Protein %	Fat %	Carbohydrate %	Ash %
*Chlorella vulgaris*	10.75 ± 0.37	37.61 ± 1.38	3.61 ± 0.32	41.55 ± 1.03	6.48 ± 1.40

### 3.3 Amino acid and β-glucan content analysis

**Fig. 6:**
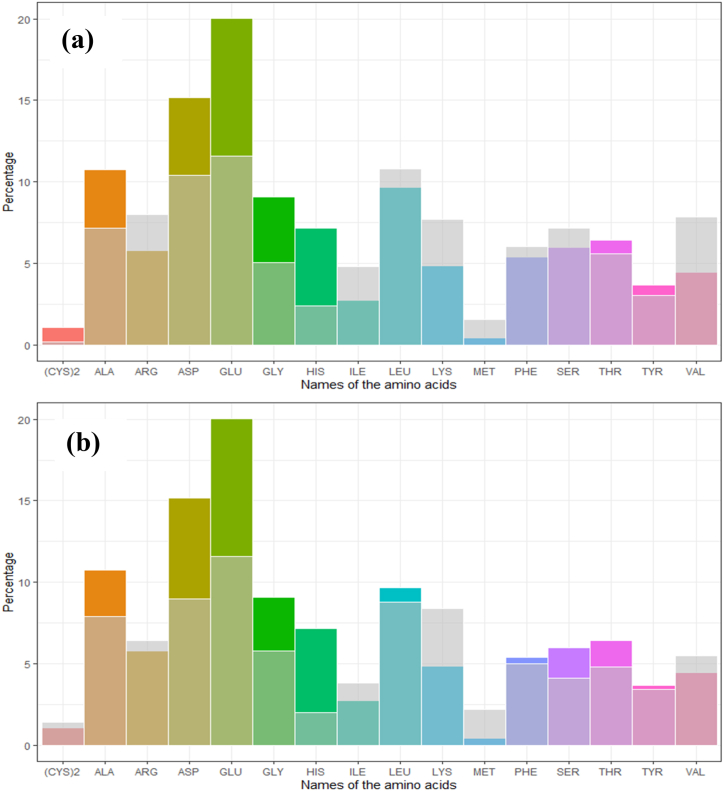
Comparative analysis of the percentage of amino acids with two different studies Beaker et al. (2006) (Fig. 6a), and Shafi et al. (2014) (Fig. 6b). Previous research is represented in grey, while datasets from current studies are displayed in multicolor. Comparatively, the percentages of Alanine, Aspartic acid, Glutamine, Glycine, and Histidine in existing specimens are significantly higher. Nonetheless, the degrees of change for the remaining amino acids vary between the two investigations.

Out of the 17 amino acids identified in the dry algae powder, nine fell into the category of essential amino acids, as indicated in Table 2. Among these 17 amino acids, glutamic acid had the highest concentration at 17.7 %, while Methionine had the lowest concentration at 0.4 %. Leucine made the most substantial contribution to the essential amino acid profile, accounting for 8.5 %, while methionine had the smallest contribution at 0.4 %. The comparative studies also supported the higher level of glutamic acid production (Fig. 6a & b).

**Table 2:** A list describing all the amino acids present in Chlorella sp. (actual amount and relative distributions) comparison to other studies.

Compound Name	Amount [mg/g]	Amount [g/100g]	other research [g/100g] (a) [5]	other research [g/100g] (b) [2]	Recommendation from FAO/WHO [g/100g] [60]
ASP	15.167 ± 0.29	13.4	10.39	9.0	N/A
THR	6.399 ± 0.04	5.7	5.60	4.8	4.00
SER	5.997 ± 0.07	5.3	7.17	4.1	N/A
GLU	20.034 ± 0.17	17.7	11.6	11.6	N/A
GLY	9.071 ± 0.06	8.0	5.05	5.8	N/A
ALA	10.743 ± 0.19	9.5	7.18	7.9	N/A
(CYS)2	1.043 ± 1.14	1.2	0.18	1.4	3.50
VAL	4.46 ± 0.08	3.9	7.86	5.5	5.00
MET	0.424 ± 0.63	0.4	1.55	2.2	N/A
ILE	2.733 ± 0.07	2.4	4.82	3.8	4.00
LEU	9.66 ± 0.08	8.5	10.78	8.8	7.00
TYR	3.667 ± 0.42	3.2	3.02	3.4	6.00
PHE	5.387 ± 0.25	4.8	6.02	5.0	N/A
HIS	7.167 ± 1.86	6.3	2.40	2.0	N/A
LYS	4.859 ± 0.05	4.3	7.70	8.4	5.50
ARG	5.776 ± 0.09	5.1	7.97	6.4	N/A
	112.932 ± 5.9	100.0			

N/A: Not available.

In this experiment, 4.324 ± 0.001 % β-glucan was determined in dry green *C. vulgaris* powder.

### 3.4 Heavy metal concentration determination

The levels of Cadmium, Chromium, and Lead concentration was found in the permissible range (Table 3).

**Table 3:** Heavy metal status of dried algae powder.

S.No	Parameter	Concentration(mg/kg)
1	Lead (Pb)	0.0020 (<0.25 mg/kg)
2	Cadmium (Cd)	Not detected (<0.25 mg/kg)
3	Chromium(Cr)	0.28 (<0.50 mg/kg)

### 3.5 Microbe content determination

After 15 min of UV exposure, the microbial analysis revealed the following results for a 25-g sample: a Total Bacterial Count (TBC) of 1.1 × 10 CFU, a Total Yeast & Molds Count of 1.1 × 10 CFU (colony-forming units), and the absence of *Salmonella* sp. and *Shigella* sp. Additionally, Hemorragic *Escherichia coli* was not detected in the sample (Table 4). These findings indicate in reducing microbial contamination in the sample, ensuring its safety and quality.

**Table 4:** Microbial analysis of dried chlorella powder.

Microbial Count	Before UV exposing	After 15 min of UV exposing
Total Bacteria Count(TBC)	8.5 × 10 CFU	1.1 × 10 CFU
Total Yeast & Molds Count	1.3 × 10 CFU	1.1 × 10 CFU
Salmonella sp. and Shigella sp.	Not Found in 25 gm of Sample	Not Found in 25 gm of Sample
Hemorragic *E. coli*	Not Found in 25 gm of Sample	Not Found in 25 gm of Sample

## 4 Discussion

Optimal growth conditions, especially the light exposure period, are crucial in maximizing biomass production of *C. vulgaris.* Indoor phyto-tanks provide a controlled environment where factors such as temperature, light intensity, pH, and nutrient availability can be manipulated to enhance growth. Several studies have recently concentrated on developing more efficient phytoplankton growing techniques to increase the biomass of microalgae and the nutritional value of these organisms [[27], [28], [29], [30], [31]]. Despite significant improvements in microalgal production in recent years, the amount of biomass generated is insufficient to meet the demands of the market [29,30].

The growth rate and composition of biomass in various microalgae species are significantly influenced by the intensity of light and the duration of light exposure, making these two aspects crucial in the cultivation process [[32], [33], [34], [35], [36], [37]].

Additionally, algal biomass and algae-derived compounds have a wide range of potential applications, including as a source of natural antioxidants and bioactive compounds with antiviral, antifungal, antibacterial, and antioxidant properties [38,39]. Algae can also be used in biofilm reactors to optimize microalgae biomass production and overcome harvesting bottlenecks, leading to more efficient and sustainable production [40]. Microalgae are widely distributed and rich in nutrients, showcasing a solid ability to utilize photosynthesis [41]. The diverse nutritional components and bioactive substances found in microalgae can fulfill the nutritional needs of early-stage aquaculture fish development [42]. The objective of these initiatives is to improve the nutritional content of these microorganisms.

Several studies have explored how the duration of light exposure, or photoperiod, influences the productivity and market fluctuations of the microalga *C. vulgaris* [35]. Additionally, Chlorella comes in various strains, some of which exhibit biomass productivity spanning from 0.18 to 0.34 g per liter per day, as observed by De Greque MM and Costa JAV in 2007 and Ryu HJ et al., in 2009 [43,44]. Kim et al., 2012 reported specific growth rates and biomass productivity for Chlorella sp. in the presence of 0.04 % CO_2_, yielding 0.24 g per liter per day [45]. Interestingly, higher light intensity has been found to decrease Chlorella productivity. Ho et al., 2012 documented that six different *Chlorella* strains achieved biomass productivity ranging from 0.22 ± 0.02 to 0.44 ± 0.02 g per liter per day under continuous illumination but at a higher intensity of 140 μmol per square meter per second [46].

The continuous addition of CO_2_ (2.5 %) to algal cultures has been a common practice in previous studies and likely contributed to enhanced microalgae productivity. In contrast, our own study observed relatively low biomass productivity for *C. vulgaris* under continuous illumination without CO2 enrichment, reaching a maximum biomass concentration of 6.4833 × 10^6^ cells per milliliter in a 16-h light and 8-h dark photoperiod. Furthermore, Jacob-Lopes et al., 2009 conducted research on the growth of blue-green microalgae under various lighting cycles (0:24, 2:22, 4:20, 6:18, 8:16, 10:14, 12:12, 14:10, 16:8, 18:6, 20:4, 22:2, and 24:0),(night:day), revealing that biomass production decreased linearly as the duration of light exposure decreased [15]. Notably, our study's key finding underscores the significant influence of light exposure duration on *C. vulgaris* biomass production, with the 16-h light and 8-h dark photoperiod resulting in the highest biomass concentration at 6.48 × 10^7^ ± 0.50 cells per milliliter [15].

Depending on the species of microalgae, several studies have discovered that microalgae development can be controlled by the photoperiod as opposed to the total amount of light received each day [[47], [48], [49], [50]]. This suggests that longer daily exposure to light promotes more efficient photosynthesis and, consequently, higher biomass accumulation. In contrast, the 8:16-h light-dark photoperiod resulted in lower biomass production, emphasizing the importance of an adequate light supply for microalgal growth.The proximate analysis of *C. vulgaris* dry powder revealed important insights into its composition. Notably, the microalgae exhibited a low lipid content of 0.48 %, making it a less suitable candidate for lipid production compared to other microalgal species. Under ideal growth conditions, *C. vulgaris* can contain 5–40 % lipids in terms of dry biomass weight. These lipids primarily consist of glycolipids, waxes, hydrocarbons, phospholipids, and small quantities of free fatty acids [51]. However, when growth conditions become unfavorable, the lipid content, primarily in the form of triacylglycerols, can increase significantly, potentially reaching as high as 58 % [52].

Proteins play a pivotal role in microalgae's chemistry and makeup. They serve essential functions like cell growth, repair, and maintenance, while also acting as cellular engines, chemical messengers, controllers of cellular processes, and defenders against external threats [53]. However, the high protein content of approximately 37.61 % is a valuable characteristic, as it positions *C. vulgaris* as a potential protein source for animal feed. Previous studies show 36.16 mg/l protein content found in *C. vulgaris* biomass production in bold basal media [54].

Furthermore, the substantial carbohydrate fraction (51.16 %) enhances its suitability as a feed source, given that carbohydrates are vital for supplying energy in animal diets, aligning with earlier research where *C. vulgaris* exhibited a rapid response to nitrogen starvation, accumulating carbohydrates to a level of 51.3 % in just four days under nitrogen-deprived conditions [[50], [55]]. These results emphasize the adaptability of *Chlorella vulgaris* as a promising component in animal feed formulations, particularly within the realms of aquaculture and livestock farming.

The nutritional value of a protein is assessed based on its composition of amino acids [2]. In the case of *C. vulgaris*, a type of microalgae, its amino acid composition is notably favorable and even surpasses the recommended human nutrition standards set by the World Health Organization (WHO) and the Food and Agricultural Organization (FAO). This is due to the fact that *C. vulgaris* cells have the capability to produce both essential and non-essential amino acids. Amino acid analysis revealed the presence of nine essential amino acids in *Chlorella vulgaris*, with glutamic acid being the most abundant at 17.7 %. The comparative analysis of amino acid percentage revealed that this strain produced higher levels of aspartic acid and glutamic acid (Fig. 6a & b). Essential amino acids are crucial for animal growth and health, and the presence of these amino acids in *Chlorella vulgaris* further supports its potential as a high-quality protein source for animal nutrition.

(β-glucan) is a dietary fiber commonly found in oat and barley bran, serving as an economical byproduct of milling. It is frequently added to food products with the aim of delivering health advantages [56]. Primarily, β-glucans are situated within the inner aleurone and subaleurone cell walls of these grains. In contrast, other cereals contain β-glucan but in notably smaller quantities; for instance, sorghum contains 1.1–6.2 g, rye 1.3–2.7 g, maize 0.8–1.7 g, triticale 0.3–1.2 g, wheat 0.5–1.0 g, durum wheat 0.5–0.6 g, and rice 0.13 g of β-glucan [57]. Conversely, in *C. vulgaris*, an estimated 4.32 g of β-glucan is present, surpassing these quantities, suggesting its potential use as a valuable nutritional source.

The microbiological analysis of the *C. vulgaris* biomass showed low microbial contamination levels, with no detection of Salmonella sp., Shigella sp., or Hemorrhagic *E. coli*. This indicates that the cultivated biomass is safe and of good quality for use as a feed source according to the International Microbiological Standard recommendations [58]. Furthermore, an analysis was conducted to determine the levels of heavy metals, with a particular focus on Cadmium (Cd), Chromium (Cr), and Lead (Pb) in the powdered sample. Additionally, Mottalib et al. (2016) reported a notable presence of heavy metals in the atmosphere. Regardless, the findings indicated that the concentrations of these heavy metals fell within acceptable limits and remained within typical ranges [59].

The results of this study have practical implications for the commercial cultivation of *C. vulgaris*. Optimizing the light exposure period, as demonstrated by the 16:8-h light-dark photoperiod, can lead to increased biomass production. This efficiency can contribute to the cost-effectiveness of microalgae cultivation for various applications, including animal feed and the production of bioactive compounds.

## 5 Conclusion

In conclusion, this study focuses on the role of light exposure periods in the cultivation of *C. vulgaris* and its potential applications as a feed source. The 16:8-h light-dark photoperiod was found to be the most effective in promoting biomass production. The microalga's dry powder composition, including its high protein content, essential amino acids, and low microbial and heavy metal contamination, makes it a valuable resource for the aquaculture and livestock industries. Further research and commercial-scale cultivation efforts can harness the potential of *C. vulgaris* for sustainable animal nutrition and other biotechnological applications.

## Funding

Funds for this work were received from the project of “Establishment of Research Facilities on Processing of Safe and Healthy Dry Fish and Indoor Farming at 10.13039/501100005999BCSIR Centre Dhaka and Chattogram”, Ministry of Science and Technology, Bangladesh.

## CRediT authorship contribution statement

**Turfatul Jannat Jui:** Writing – original draft, Validation, Methodology, Formal analysis, Conceptualization. **Anika Tasnim:** Writing – review & editing, Writing – original draft, Visualization, Validation, Methodology. **S.M. Rashadul Islam:** Methodology. **Omar Hamza Bin Manjur:** Visualization, Formal analysis. **Md. Saddam Hossain:** Methodology. **Nishat Tasnim:** Supervision. **Debabrata Karmakar:** Supervision. **Md. Rakibul Hasan:** Supervision, Investigation, Funding acquisition, Conceptualization. **Md. Rezaul Karim:** Supervision, Project administration, Investigation, Funding acquisition.

## Declaration of competing interest

The authors declare that they have no personal relationships or known competing financial interests that could have appeared to influence the work reported in this paper.
